# Research on Constitutive Modeling of DH460 Continuous Casting Steel with the Solidification End Reduction Process

**DOI:** 10.3390/ma18020453

**Published:** 2025-01-20

**Authors:** Bochun Liang, Chunxi Han, Tan Zhao, Cheng Ji, Miaoyong Zhu

**Affiliations:** 1 State Key Laboratory of Metal Material for Marine Equipment and Application, Angang Steel Co., Ltd., Anshan 114021, China; 2 School of Metallurgy, Northeastern University, Shenyang 110819, China

**Keywords:** continuous casting, heavy reduction, flow stress, constitutive model

## Abstract

The constitutive model was commonly used to describe the flow stress of materials under specific strain, strain rate, and temperature conditions. In order to study the thermal–mechanical behavior of DH460 continuous casting steel during the solidification end heavy reduction (HR) process accurately. The high-temperature compression experiment was carried out, and phenomenological constitutive models were established based on the experimental results. A new strain-strengthening factor (*D*(*ε*)) was proposed in order to improve the prediction accuracy of the current constitutive models. Then, the further-modified models were established. It was found that the new strain-strengthening factor significantly reduced the error of models. The average relative error (*AARE*) of the further-modified Johnson–Cook model and the further-modified Zerilli–Armstrong model were 6.27% and 5.54%, respectively. The results showed that the further-modified models were more suitable for describing the constitutive behavior of DH460 continuous casting steel during the solidification end reduction.

## 1. Introduction

The solidification end reduction of continuous casting steel has the characteristics of a large temperature span, a high strain rate, and an obvious microstructure difference [1]. Under the condition of high strain rate, dynamic recrystallization softening, dynamic recovery, and work hardening will occur. As one of the key performance indexes in hot working deformation, flow stress can comprehensively reflect the hardening and softening processes of materials. It is necessary to accurately characterize the constitutive model of continuous casting steel in order to accurately reveal the thermodynamic influence during the reduction deformation of solidification ends. The constitutive model describes the influence of temperature, strain rate, and strain on the heat flow behavior of the material in the thermal machining process, which is essential for the finite element simulation, design and control of the thermal machining process. There is a complex nonlinear relationship between the flow stress and the parameters of hot working due to the simultaneous occurrence of work hardening and softening in the process of hot working. Therefore, in order to more accurately describe this relationship, several constitutive models have been explored by researchers, which can be classified into three main categories [2]: phenomenological constitutive models, physical-based constitutive models, and artificial neural network models [3,4].

Common phenomenological constitutive models include the Johnson–Cook model (J-C model), Fields–Backofen model (F-B model), Zerilli–Armstrong model (Z-A model), and Arrhenius model. The J-C model was first introduced into the finite element analysis because of its simple parameter form and convenient calculation [5]. Later, Lin et al. proposed a modified J-C model considering the coupling effects of strain, strain rate, and deformation temperature to describe the tensile behavior of common alloy steels. Most of the subsequent studies on the accuracy correction of the J-C model were based on this revised model [6,7,8]. Samantaray et al. made important modifications to the Z-A model, which consider the effects of thermal softening, strain rate hardening, and isotropic hardening, as well as the coupling effects of strain, strain rate, and temperature on flow stress [9]. The high-temperature flow behavior of D9 alloy in the specified hot working region was predicted successfully [10]. The subsequent modified models [11] were also mostly based on the model proposed by Samantaray. Shokry et al. modified the J-C model and the Z-A model when they tried to fit the contract with multivariate quadratic polynomials. This modification improved the applicability of the two models, which could accurately predict most alloys and steels, as well as some plastic materials [12,13]. The Arrhenius model was first proposed by Sellars and Tegart to describe and predict the thermal deformation behavior of materials [14]. Pu et al. modified the relationship between the parameters of the model and the strain by polynomial fitting and predicted the high-temperature constitutive relationship of Al-Ti alloy more accurately [15]. Dai et al. proposed a strain-compensated Arrhenius model to accurately represent the flow behavior of 5083 aluminum alloy during thermal compression [16]. Wang et al. modified the Arrhenius equation by including the liquid fraction to predict the peak stress of 6063 aluminum alloy during semi-solid compression [17].

In this paper, DH460 continuous casting steel was used as the experimental specimens for high-temperature compression experiments. The simulated temperature range was 900 °C to 1300 °C, and the strain rates were 0.001 s^−1^, 0.01 s^−1^, and 0.1 s^−1^, respectively. Based on experimental data, the modified J-C model, the modified Z-A model, and the Arrhenius model were used to model the constitutive relationship. Moreover, in this study, a new strain-strengthening factor was proposed to further improve the prediction accuracy of the J-C model and Z-A model. The prediction accuracy of all models was then compared and analyzed. The results showed that the further-modified constitutive model captured the recrystallization during the deformation process effectively, with higher accuracy at lower strains. The further-modified Z-A model obtained the highest accuracy of all the constitutive models and provided a good description of the constitutive behavior of the metal in DH460 continuous cast steel during the reduction process at the solidification end.

## 2. Materials and Methods

**Figure 1:**
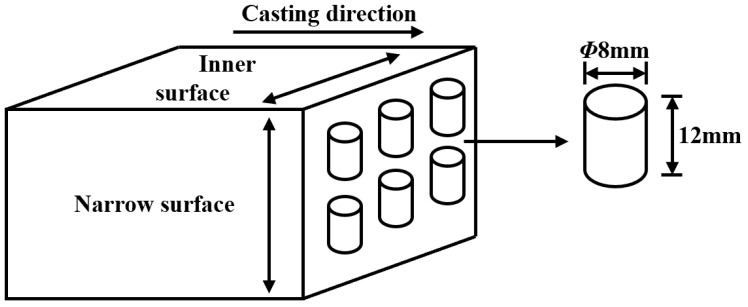
Sampling locations of the compressed sample on DH460.

Samples for the experiment were obtained from DH460 continuous casting steel produced by a domestic steel plant, with the chemical composition detailed in Table 1. The sampling direction was perpendicular to the drawing direction, avoiding the central area of center segregation and more serious porosity. Figure 1 illustrates that the samples were wire-cut and processed into cylindrical specimens with a diameter of 8 mm and a length of 12 mm.

**Figure 2:**
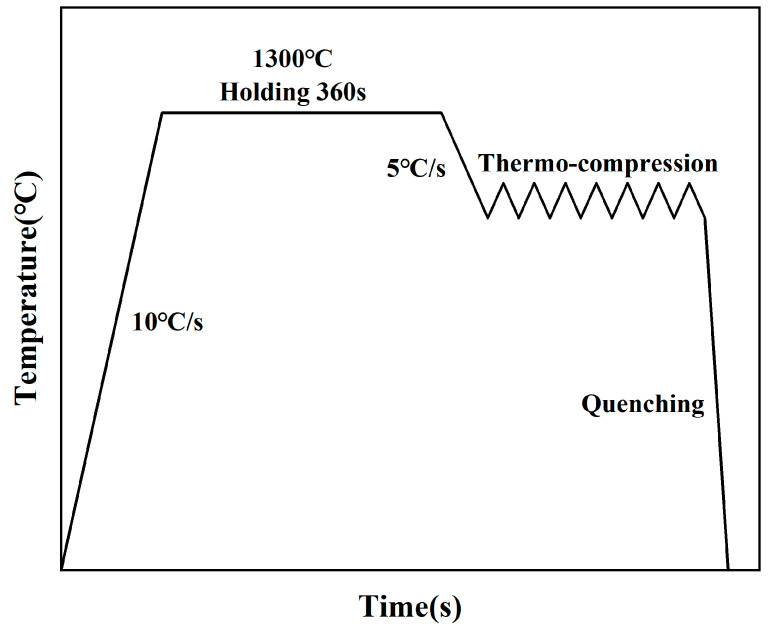
Schematic representation of the thermal compression experimental procedure.

Thermal compression experiments were conducted on the THERMECMASTOR-Z100KN thermal simulation testing machine (Tokyo, Japan), with the experimental temperature range set between 900 and 1300 °C at intervals of 50 °C. Three strain rates were selected for 0.1 s^−1^, 0.01 s^−1^, and 0.001 s^−1^. In the isothermal compression tests, specimens were first heated to 1300 °C at a rate of 10 °C/s. The specimens were held for 360 s to ensure temperature uniformity. The specimens were subsequently cooled to the compression temperature at a rate of 5 °C/s. Eventually, the specimens were subjected to compression until a true strain of 0.7 was reached under constant temperature and strain rate conditions. Figure 2 provides an overview of the experimental process.

**Figure 3:**
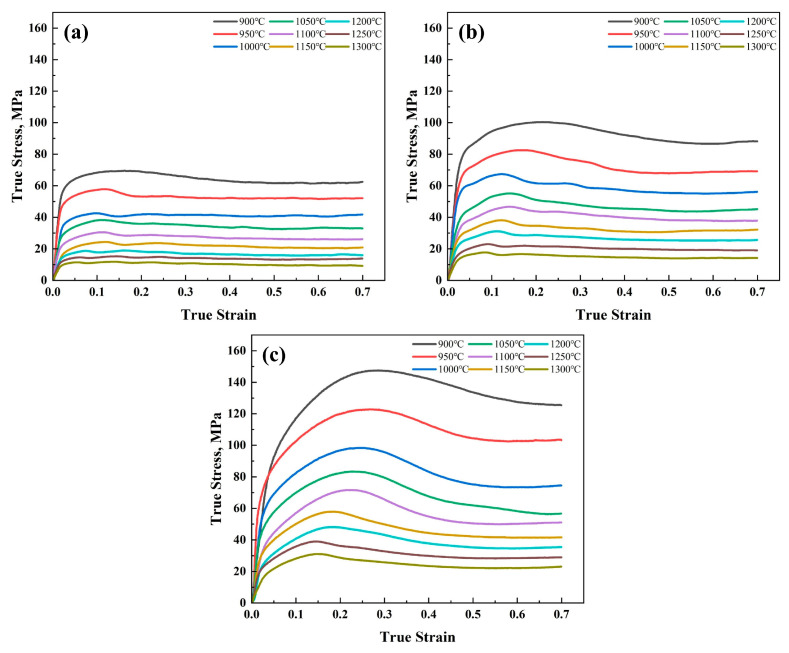
True stress–strain relationships of DH460 under compressive deformation: (**a**) 0.001 s^−1^, (**b**) 0.01 s^−1^, and (**c**) 0.1 s^−1^.

The true stress–strain curves of DH460 continuous casting steel at different temperatures and different strain rates are shown in Figure 3. At low strain levels, the metal’s work-hardening effect dominates over the softening effect of dynamic recovery, resulting in a rapid increase in stress with strain. When the strain reaches critical strain, dynamic recrystallization takes place, leading to the formation of recrystallized grains, which help to slow the rate of crystal energy increase and decrease dislocation density [18]. The stress peaks when the softening effect is equal to the work-hardening effect. As the strain continues to increase, the internal energy of the crystal continues to increase, and the dynamic recrystallization occurs more obviously [19]. At this point, the softening effect is stronger than the work-hardening effect, and the stress decreases with increasing strain. Finally, the stress remains stable, at which point the softening effect reaches a dynamic equilibrium with the work-hardening effect.

## 3. Establishment of Constitutive Models

### 3.1. Johnson–Cook-Type Model

The Johnson–Cook model was derived by Johnson and Cook on the basis of the Hopkinson tension test and other experimental data. The model was simple in structure and easy to determine or fit the parameters. The basic form of the J-C model can be expressed as follows:

(1) $$\sigma =\left[A+B\varepsilon^{n}\right]\left[1+C\ln\left(\frac{\dot{\varepsilon }}{{\dot{\varepsilon }}_{0}}\right)\right]\left[1-{\left(\frac{T-T_{r}}{T_{m}-T_{r}}\right)}^{m}\right]$$

where, *A*, *B*, *C*, *n*, and *m* are material parameters; *σ* is stress; *ε* is strain; $\varepsilon_{0}$ is the reference strain; $\dot{\varepsilon }$ is the strain rate; ${\dot{\varepsilon }}_{0}$ is the reference strain rate; *T* is the temperature; *T_r_* is the reference temperature; and *T_m_* is the melting point temperature.

**Figure 4:**
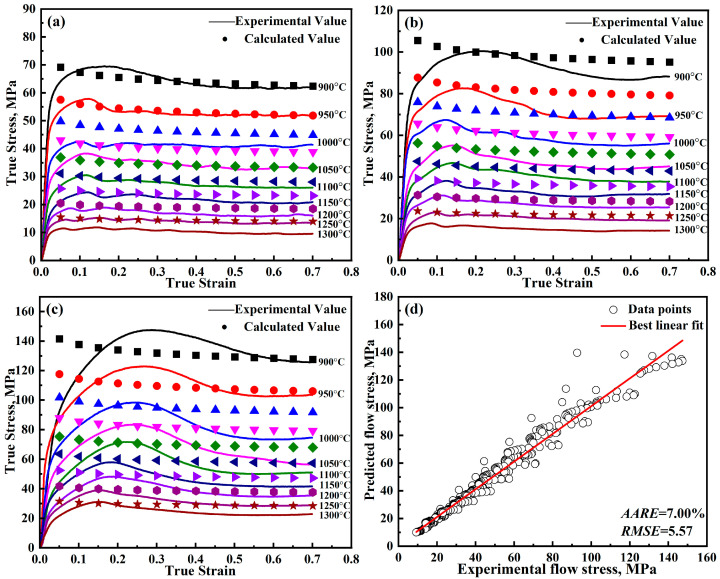
Predicted and experimental results of the modified J-C model for DH460 at various strain rates: (**a**) 0.001 s^−1^, (**b**) 0.01 s^−1^, (**c**) 0.1 s^−1^, and (**d**) error analysis.

Although the parameters of the J-C model are simple and easy to calculate, its accuracy is low, and its applicability is limited [20]. Lin et al. modified the J-C model by considering the coupling effects of strain, strain rate, and deformation temperature, which can accurately estimate the flow stresses of typical high-strength alloy steels. The basic form of the modified J-C model can be expressed as follows:

(2) $$\sigma =\left[A_{1}+B_{1}\varepsilon +B_{2}\varepsilon^{2}\right]\left(1+C_{1}\cdot \ln{\dot{\varepsilon }}^{*}\right)\exp\left[-\left(\lambda_{1}+\lambda_{2}\ln{\dot{\varepsilon }}^{*}\right)\cdot T^{*}\right]$$

where, *A*_1_, *B*_1_, *B*_2_, *C*_1_, *λ*_1_, and *λ*_2_ represent material parameters and unknown coefficients, which can be determined through fitting experimental data; ${\dot{\varepsilon }}_{0}$ is the reference strain rate; *T_r_* is the reference temperature; ${\dot{\varepsilon }}^{*}=\frac{\dot{\varepsilon }}{{\dot{\varepsilon }}_{0}}$; and $T^{*}=T-T_{r}$. The modified J-C is solved using the least squares method [21,22,23]. The parameters obtained from the model are presented in Table 2, while the detailed outcomes are illustrated in Figure 4.

As strain increases, the softening effects of dynamic recovery and dynamic recrystallization progressively balance the work hardening, leading to the peak stress. The modified J-C model cannot capture the recrystallization behavior during metal deformation, so the prediction error is large. In view of this, this paper proposes a new strain-strengthening factor to further modify the J-C model. The basic form of *D*(*ε*) is as follows:

(3) $$D\left(\varepsilon \right)=\sum_{i=0}^{6}D_{i}\varepsilon^{i}$$

**Figure 5:**
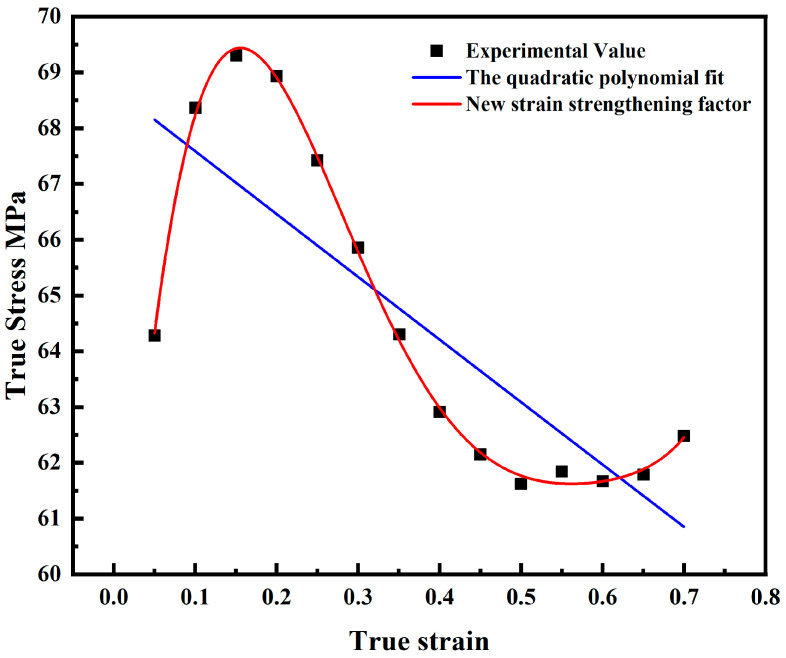
Comparison of the effect of the new strain-strengthening factor and the original strain-strengthening term.

The comparison of the effect of the new strain-strengthening factor and the original factor is shown in Figure 5. As can be seen from the figure, the new strain-strengthening factor fits the experimental value much better. The expression of the further-modified J-C model can be written as follows:

(4) $$\sigma =D\left(\varepsilon \right)\left(1+C_{1}\cdot \ln{\dot{\varepsilon }}^{*}\right)\exp\left[-\left(\lambda_{1}+\lambda_{2}\ln{\dot{\varepsilon }}^{*}\right)\cdot T^{*}\right]$$

The further-modified J-C model comprises three components: $f\left(\varepsilon \right)=D\left(\varepsilon \right)$ accounts for strain strengthening; $f\left(\dot{\varepsilon }\right)=\left(1+C_{1}\cdot \ln{\dot{\varepsilon }}^{*}\right)$ represents strain rate strengthening; and $f\left(T,\dot{\varepsilon }\right)=\exp\left[-\left(\lambda_{1}+\lambda_{2}\ln{\dot{\varepsilon }}^{*}\right)\cdot T^{*}\right]$ describes thermal softening, which reflects both the temperature-induced softening of stress and the coupled influence of temperature and strain rate on stress.

When the temperature *T* is set to the reference value (900 °C) and the strain rate is equal to the reference value (0.001 s^−1^), Equation (4) simplifies to Equation (5). This paper utilizes the stress–strain data obtained from a thermal compression experiment and solves for the parameters *D*_0_–*D*_6_ using the polynomial fitting method. The result is shown by the red line in Figure 5, and the values of *D*_0_–*D*_6_ are displayed in Table 3.

(5) $$\sigma =D\left(\varepsilon \right)$$

When the temperature *T* is set to the reference value (900 °C), Equation (4) can be abbreviated as:

(6) $$\sigma =D\left(\varepsilon \right)\left(1+C_{1}\cdot \ln{\dot{\varepsilon }}^{*}\right)$$

Transfer the items to sort out:

(7) $$C_{1}\cdot \ln{\dot{\varepsilon }}^{*}=\frac{\sigma }{D\left(\varepsilon \right)}-1$$

**Figure 6:**
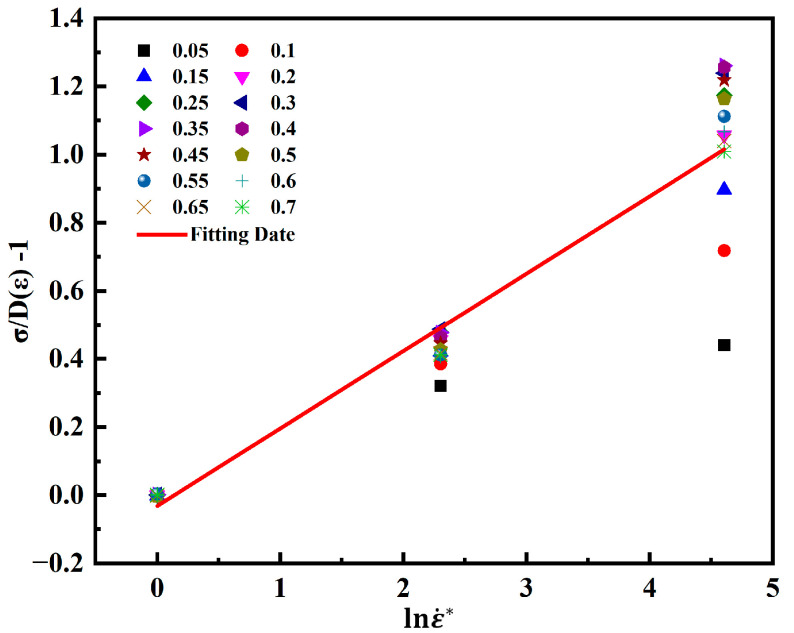
Least squares method to solve parameter *C*_1_.

According to Equation (7), *C*_1_ is the slope of the function $\left(\frac{\sigma }{D\left(\varepsilon \right)}-1\right)-\ln{\dot{\varepsilon }}^{*}$. The parameter *C*_1_ is calculated as shown in Figure 6.

By substituting obtained parameters into Equation (4), and in order to calculate $\lambda_{1}$ and $\lambda_{2}$, we introduce $\lambda =\lambda_{1}+\lambda_{2}\ln{\dot{\varepsilon }}^{*}$. The following equation can be obtained:

(8) $$\ln\left(\frac{\sigma }{D\left(\varepsilon \right)\left(1+C_{1}\cdot \ln{\dot{\varepsilon }}^{*}\right)}\right)=-\lambda \cdot T^{*}$$

**Figure 7:**
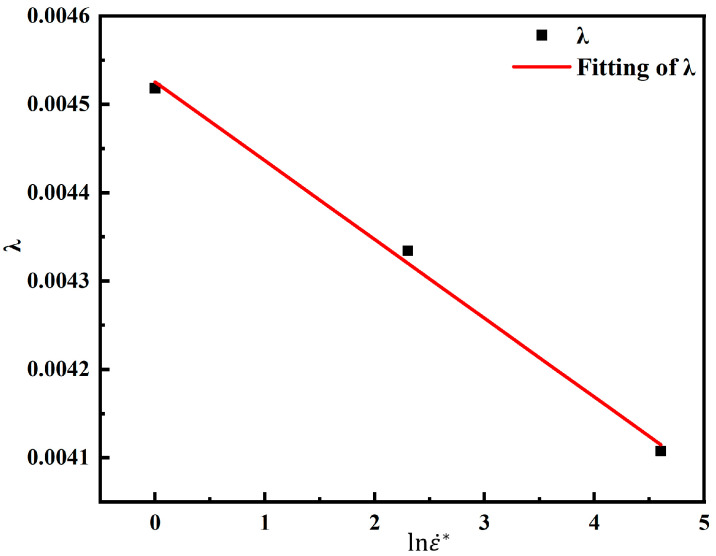
Least squares method for solving parameters $\lambda_{1}$ and $\lambda_{2}$.

With the strain rate unchanged, the stress values at different temperatures and strain conditions are substituted into Equation (8). The correspondence of $\ln{\dot{\varepsilon }}^{*}$ and λ can be obtained. As shown in Figure 7, the slope of the function $\lambda =\lambda_{1}+\lambda_{2}\ln{\dot{\varepsilon }}^{*}$ is $\lambda_{2}$ and the intercept is $\lambda_{1}$.

**Figure 8:**
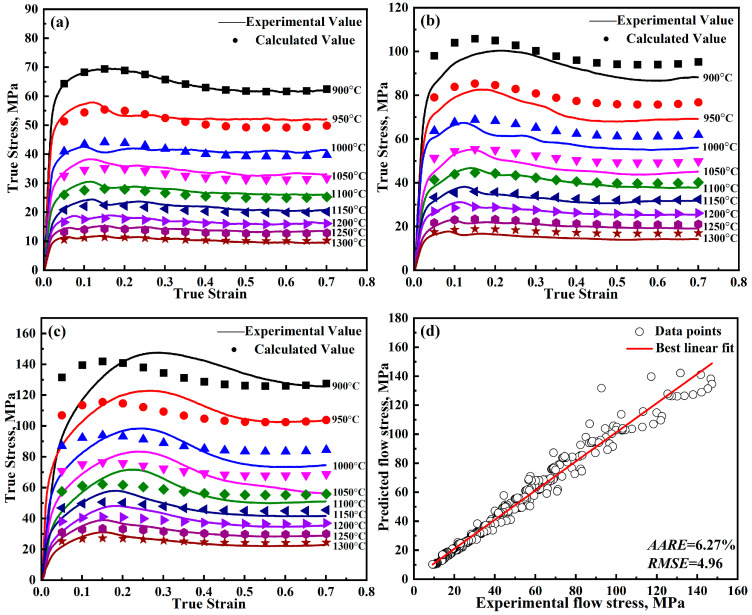
Predicted and experimental results of the further-modified J-C model for DH460 at various strain rates: (**a**) 0.001 s^−1^, (**b**) 0.01 s^−1^, (**c**) 0.1 s^−1^, and (**d**) error analysis.

Now that all the parameters have been solved, and Table 3 lists the parameters for the further-modified J-C model. The detailed results are shown in Figure 8. From Figure 4 and Figure 8, it can be found that the predicted curves of the modified J-C model only show a decreasing trend. While the predicted curves of the further-modified J-C model show a first increasing, then decreasing, and finally smooth trend, which is in better agreement with the experimental values.

### 3.2. Zerilli–Armstrong-Type Model

The Zerilli–Armstrong model was proposed by Zerilli and Armstrong in 1987 and was divided into two kinds of equations according to the type of metal lattice structure: face-centered cubic and body-centered cubic. The basic forms of the Z-A model are shown below.

(9) $$\sigma =E_{0}+E_{2}\varepsilon^{\frac{1}{2}}\exp\left(-E_{3}+E_{4}T\cdot \ln\dot{\varepsilon }\right)$$

(10) $$\sigma =E_{0}+E_{1}\exp\left(-E_{3}+E_{4}T\cdot \ln\dot{\varepsilon }\right)+E_{5}\varepsilon^{n}$$

Due to the limitations of the Z-A model, it cannot meet the requirements of the conditions of solidification-end reduction, so the simple Z-A model is not used to describe the metal constitutive behavior of continuous casting steel.

**Figure 9:**
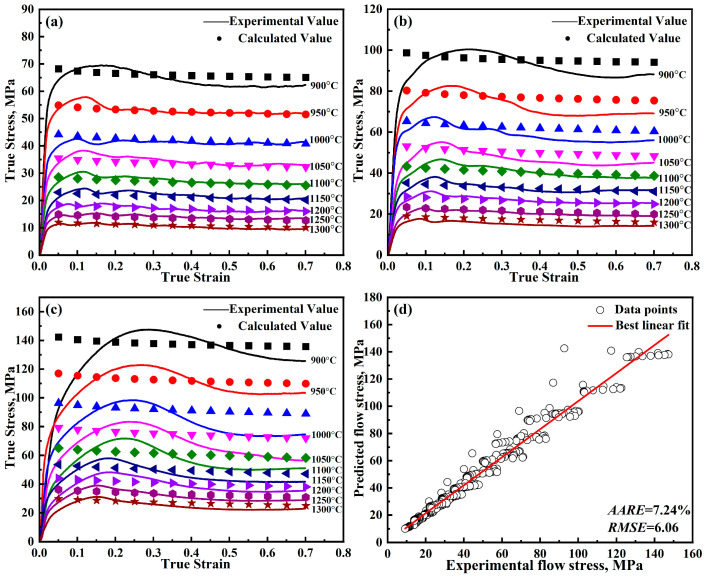
Predicted and experimental results of the modified Z-A model for DH460 at various strain rates: (**a**) 0.001 s^−1^, (**b**) 0.01 s^−1^, (**c**) 0.1 s^−1^, and (**d**) error analysis.

The modified Z-A model proposed by Samantaray et al. was one of the important modifications of the Z-A model. It incorporates not only the effects of temperature, strain, and strain rate on stress but also accounts for the coupling influence of temperature and strain rate, as well as temperature and strain on stress. The basic form of the modified Z-A model is as follows:

(11) $$\sigma =\left[E_{1}+E_{2}\varepsilon^{n}\right]\exp\left[-\left(E_{3}+E_{4}\varepsilon \right)\cdot T^{*}+\left(E_{5}+E_{6}T^{*}\right)\cdot \ln{\dot{\varepsilon }}^{*}\right]$$

where *E*_1_~*E*_6_ are model parameters, and the other variables have the same meaning as the model shown before. In this modified model, the parameters *E*_1_, *E*_2_, and *n* represent the strain hardening term; *E*_3_ and *E*_4_ represent the softening term; and *E*_5_ and *E*_6_ constitute the strain rate term. The procedure for determining the parameters of the modified Z-A model is outlined in previous studies [24,25], with the corresponding results provided in Table 4. The model predictions are shown in Figure 9.

As can be seen in Figure 9, the modified Z-A model, like the modified J-C model, shows a single overall decreasing trend and fails to capture the dynamic recrystallization behavior during metal deformation. Hence, introducing the new strain-strengthening factor into the Z-A model leads to Equation (12). The parameters of the further-modified Z-A model are fitted using the same methodology as in Section 3.1.

(12) $$\sigma =D\left(\varepsilon \right)\exp\left[-\left(E_{3}+E_{4}\varepsilon \right)\cdot T^{*}+\left(E_{5}+E_{6}T^{*}\right)\cdot \ln{\dot{\varepsilon }}^{*}\right]$$

When the strain rate is set to the reference value (0.001 s^−1^), Equation (12) can be abbreviated as:

(13) $$\sigma =D\left(\varepsilon \right)\exp\left[-\left(E_{3}+E_{4}\varepsilon \right)\cdot T^{*}\right]$$

Taking the logarithm of both sides of Equation (13) leads to Equation (14):

(14) $$\ln\sigma =\ln D\left(\varepsilon \right)-\left(E_{3}+E_{4}\varepsilon \right)\cdot T^{*}$$

**Figure 10:**
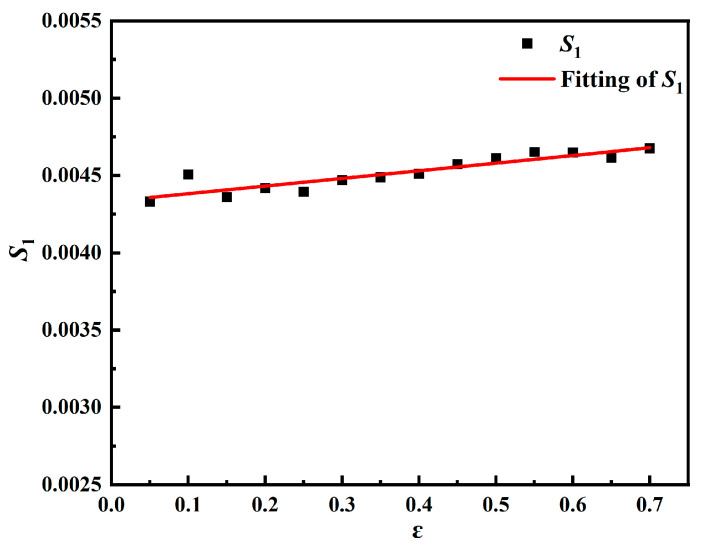
Least squares fitting process for parameters *E*_3_ and *E*_4_.

In Figure 10, *E*_3_ and *E*_4_ can be obtained according to the functional relationship of parameters *S*_1_ and ε.

Taking the logarithm of both sides of Equation (12) leads to Equation (15):

(15) $$\ln\sigma =\ln D\left(\varepsilon \right)-\left(E_{3}+E_{4}\varepsilon \right)\cdot T^{*}+\left(E_{5}+E_{6}T^{*}\right)\cdot \ln{\dot{\varepsilon }}^{*}$$

(16) $$S=E_{5}+E_{6}T^{*}$$

**Figure 11:**
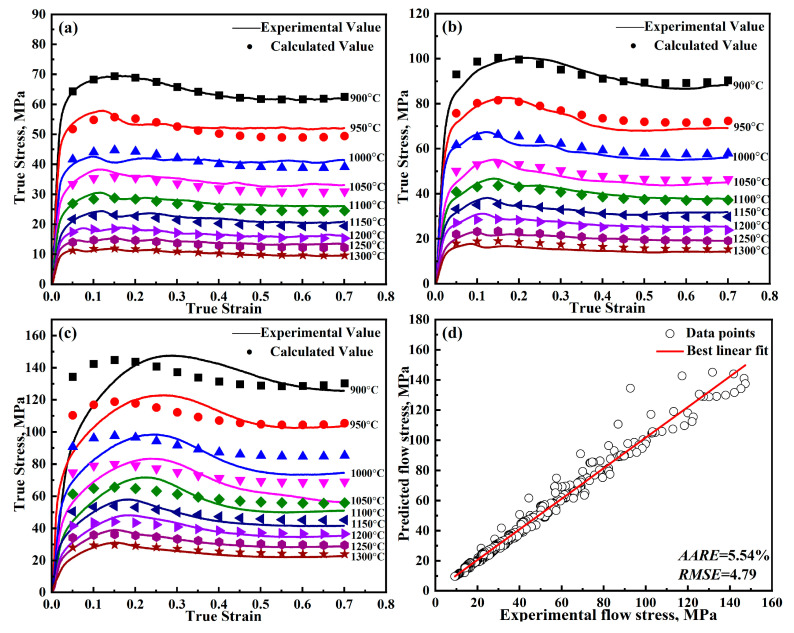
Predicted and experimental results of the further-modified Z-A model for DH460 at various strain rates: (**a**) 0.001 s^−1^, (**b**) 0.01 s^−1^, (**c**) 0.1 s^−1^, and (**d**) error analysis.

Let the slope of the function $\ln\sigma -\ln{\dot{\varepsilon }}^{*}$ be *S*, and *E*_6_ can be obtained from the slope of the function $S-T^{*}$, and *E*_5_ can be obtained from the longitudinal intercept. Different strains correspond to different groups of *E*_5_ and *E*_6_ values, and the group of *E*_5_ and *E*_6_ values with the smallest error is selected. The parameter values of the further-modified Z-A model calculated according to the experimental results are shown in Table 5. The model predictions are shown in Figure 11. As shown in Figure 9 and Figure 11, the further-modified Z-A model provides a better fit to the experimental values, and the dynamic recrystallization behavior during deformation can be well captured.

### 3.3. Arrhenius Model

In the process of the hot compression experiment, the metal microstructure corresponding to different temperature curves is also different. Since the Arrhenius model contains thermal deformation activation energy (*Q*), it can describe the difficulty of plastic deformation of metal. Therefore, the Arrhenius model can directly reflect the influence of temperature and strain rate on stress, and it was used to determine the material constants in many works of metal thermal processing properties [26,27]. The Arrhenius model exists in three distinct forms: exponential form, power exponential form, and hyperbolic sine function, according to different stress levels. Its basic form is expressed as follows:

(17) $$\dot{\varepsilon }=A\exp\left(-\frac{Q}{RT}\right)F\left(\sigma \right)$$

where *F*(*σ*) denotes the stress function, which is given by the following expression:

(18) $$F\left(\sigma \right)=\left\{\begin{array}{cc} \sigma^{n_{1}} & \alpha \sigma <0.8\\ \exp\left(\beta \sigma \right) & \alpha \sigma >1.2\\ {\left[\sinh\left(\alpha \sigma \right)\right]}^{n} & For\;all\;\sigma \end{array}\right.$$

where *R* is the ideal gas constant; *T* is the absolute temperature; *Q* is the deformation activation energy; *n* is the material stress index; and *A*, *α*, *β*, and *n*_1_ are material constants (α = *β*/*n*_1_).

The equations of low stress level and high stress level in the Arrhenius model can be regarded as the equations obtained after Taylor expansion of the hyperbolic sinusoidal function according to stress state incongruence. The *Q* in the equation is a physical quantity that represents the difficulty of rearrangement and combination of microscopic atoms in the process of thermal deformation. Its value is affected by many factors such as chemical composition, structure, deformation rate, and deformation temperature of the material.

The Arrhenius model also uses the Zenner–Hollomon factor to describe the effect of strain rate and temperature on deformation behavior. The Zenner–Hollomon factor is an important parameter in the study of flow stress and dynamic softening behavior. Its form is as follows:

(19) $$Z=\dot{\varepsilon }\exp\left(\frac{Q}{RT}\right)$$

For all stress states, Equation (20) can be obtained from Equations (17)–(19):

(20) $$Z=A\cdot {\left[\sinh\left(\alpha \sigma \right)\right]}^{n}$$

The connection between Z and flow stress can be derived from Equations (19) and (20):

(21) $$\sigma =\frac{1}{\alpha }\ln\left\{{\left(\frac{Z}{A}\right)}^{\frac{1}{n}}+{\left[{\left(\frac{Z}{A}\right)}^{\frac{2}{n}}+1\right]}^{\frac{1}{2}}\right\}$$

The material parameters *Q*, *A*, *n*, and *α* corresponding to different strains in the temperature range of 900~1300 °C are calculated, and the strains are selected as 0.05, 0.1, 0.15, 0.2, 0.25, 0.3, 0.35, 0.4, 0.45, 0.5, 0.55, 0.6, 0.65, and 0.7. Table 6 shows the material parameters corresponding to different strains.

The analysis of Table 6 reveals that the correlation between material parameters and corresponding strains is discrete and discontinuous. During the finite element simulation process, the parameters need to be continuously changed. Therefore, a polynomial fitting approach is employed to establish the functional correlation between strain and the parameters *Q*, *A*, *n*, and *α* to solve the complex nonlinear interaction between strain and material properties. According to the calculation, it is found that the accuracy is highest when the sixth-degree polynomial is used for fitting, as shown in Equation (22).

(22) $$\left\{\begin{array}{c} Q=B_{0}+B_{1}\varepsilon^{1}+B_{2}\varepsilon^{2}+B_{3}\varepsilon^{3}+B_{4}\varepsilon^{4}+B_{5}\varepsilon^{5}+B_{6}\varepsilon^{6}\\ \ln A=C_{0}+C_{1}\varepsilon^{1}+C_{2}\varepsilon^{2}+C_{3}\varepsilon^{3}+C_{4}\varepsilon^{4}+C_{5}\varepsilon^{5}+C_{6}\varepsilon^{6}\\ n=D_{0}+D_{1}\varepsilon^{1}+D_{2}\varepsilon^{2}+D_{3}\varepsilon^{3}+D_{4}\varepsilon^{4}+D_{5}\varepsilon^{5}+D_{6}\varepsilon^{6}\\ \alpha =E_{0}+E_{1}\varepsilon^{1}+E_{2}\varepsilon^{2}+E_{3}\varepsilon^{3}+E_{4}\varepsilon^{4}+E_{5}\varepsilon^{5}+E_{6}\varepsilon^{6} \end{array}\right.$$

**Figure 12:**
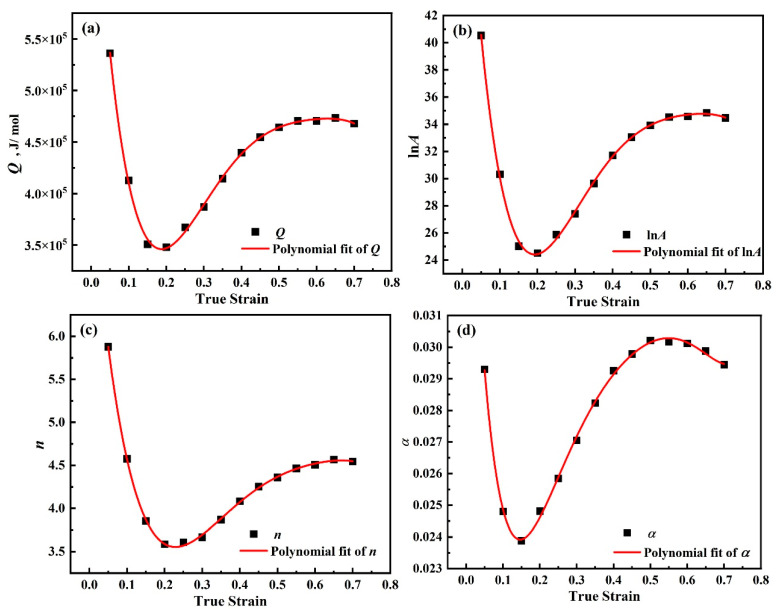
Polynomial fit between the parameters and strain of DH460 in the temperature range of 900~1300 °C. (**a**) *Q*, (**b**) ln*A*, (**c**) *n*, and (**d**) *α*.

Figure 12 shows the variation of material parameters with strain in the temperature range of 900~1300 °C.

It can be found from Figure 12 that material parameters have an obvious variation trend with strain, and using polynomial fitting to establish the relationship between material parameters and strain is considered suitable. Table 7 shows the parameters of the Arrhenius model.

**Figure 13:**
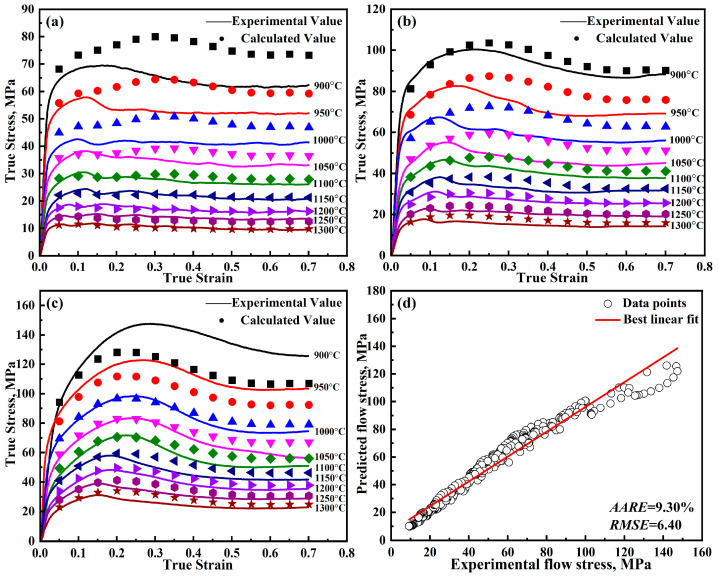
Predicted and experimental results of the Arrhenius model for DH460 at various strain rates: (**a**) 0.001 s^−1^, (**b**) 0.01 s^−1^, (**c**) 0.1 s^−1^, and (**d**) error analysis.

By substituting different strain values into Equation (22), the corresponding material parameters can be calculated. The combination of Equations (19) and (21) can calculate the predicted value of the Arrhenius model. Figure 13 illustrates the comparison of the predicted and experimental results.

## 4. Results and Discussion

In this part, the calculation results of all the above constitutive models are compared to show the improvement effect of the new strain-strengthening factor. The average relative error (*AARE*) is introduced to evaluate the accuracy of the flow stress predicted by the constitutive models at different strain rates and temperatures. The *AARE* is calculated as follows:

(23) $$AARE\left(\%\right)=\frac{1}{N}\sum_{i=1}^{N}\left|\frac{E_{i}-P_{i}}{E_{i}}\right|\times 100$$

where *E_i_* is the experimental value; *P_i_* is the predictive value; and *N* is the total number.

The *AARE* is used to calculate the average relative error between the predicted value and the experimental value, so it can represent the agreement between the predicted value and the experimental value. In order to illustrate the optimality of the models, a comprehensive evaluation of multiple error statistics indexes is usually required [28,29]. Hence, the root mean square error (*RMSE*) is added to illustrate the precision comparison of each model. *RMSE* is more focused on providing dimensionless error measures, which are calculated as follows:

(24) $$RMSE=\sqrt{\frac{\sum_{i=1}^{n}{\left(E_{i}-P_{i}\right)}^{2}}{N}}$$

The errors of the models can be seen in Table 8. Error analysis of the constitutive models for DH460 steel reveals that the Arrhenius model presents the highest error, with the *AARE* being 9.30%. As can be seen in Figure 13, the Arrhenius model, which takes into account the deformation activation energy, also shows peak stresses. However, the Arrhenius model does not notice that the strain point corresponding to the peak stress shifts forward with increasing temperature, which leads to its large error. The modified J-C model has an *AARE* of 7.00%, and the modified Z-A model has an *AARE* of 7.24%. The prediction accuracy of the above two models is similar. The further-modified Z-A model captures the recrystallization behavior during deformation well by introducing a new strain-strengthening factor, which leads to the highest prediction accuracy, with the *AARE* being 5.54% and the *RMSE* being 4.79 MPa.

## 5. Conclusions

In this paper, based on the experimental data of DH460 continuous casting steel, three phenomenological constitutive models were developed. A new strain-strengthening factor (*D*(*ε*)) was proposed to further modify J-C model and Z-A model. The conclusions can be summarized as follows:

(1) The stress of DH460 decreases with increasing temperature and increases with increasing strain rate. Under the combined effect of work hardening, dynamic recovery and dynamic recrystallization, the stress–strain curve first rises rapidly, then decreases slowly, and finally flattens out.
(2) A new strain-strengthening factor was proposed to further modify the J-C model and Z-A model. The problem that the models could not capture the dynamic recrystallization behavior during deformation was solved. The accuracy and scalability of the models were improved.
(3) By comparing all the constitutive models, it could be observed that the further-modified Z-A model had the highest prediction accuracy. By introducing the new strain correction factor, the AARE of the Z-A model was reduced from 7.24% to 5.54% and the RMSE from 6.06% to 4.79%. Comprehensively, the further-modified Z-A model could be suitable for predicting the flow behavior of DH460 continuous casting steel.

## Figures and Tables

**Table 1:** Elemental composition of DH460 continuous casting steel (wt.%).

C	Si	Mn	P	S	Nb	Ti	Al	N
0.95	0.15	1.48	0.023	0.005	0.027	0.015	0.03	0.0035

**Table 2:** Parameters of the modified J-C model.

Parameter	Value
*A*_1_	68.72
*B*_1_	−11.32
*B*_2_	0.12
*C*_1_	0.2275
*λ*_1_	0.00008914
*λ*_2_	0.004525

**Table 3:** Parameters of the further-modified J-C model.

Parameter	Value
*D*_0_	56.48076
*D*_1_	204.6357
*D*_2_	−1048.22
*D*_3_	1852.323
*D*_4_	−629.222
*D*_5_	−1439.83
*D*_6_	1139.685
*C*_1_	0.2275
*λ*_1_	0.00008914
*λ*_2_	0.004525

**Table 4:** Parameters of the modified Z-A model.

Parameter	Value
*E*_1_	1.390
*E*_2_	63.26
*n*	−0.018
*E*_3_	0.004332
*E*_4_	0.0004962
*E*_5_	0.1673
*E*_6_	0.0001

**Table 5:** Parameters of the further-modified Z-A model.

Parameter	Value
*D*_0_	56.48076
*D*_1_	204.6357
*D*_2_	−1048.22
*D*_3_	1852.323
*D*_4_	−629.222
*D*_5_	−1439.83
*D*_6_	1139.685
*E*_3_	0.004332
*E*_4_	0.0004962
*E*_5_	0.1673
*E*_6_	0.0001

**Table 6:** Parameters of the Arrhenius model.

Strain	*Q*/J·mol^−1^	ln*A*	*n*	*α*
0.05	536,151.0	40.54	5.879	0.029298
0.1	412,704.0	30.31	4.575	0.024803
0.15	350,773.2	25.01	3.854	0.023879
0.2	347,942.5	24.51	3.585	0.024811
0.25	367,176.2	25.87	3.607	0.025843
0.3	386,942.2	27.40	3.666	0.027055
0.35	414,476.4	29.64	3.868	0.028229
0.4	439,527.9	31.71	4.083	0.029255
0.45	454,626.5	33.05	4.252	0.029784
0.5	464,213.7	33.92	4.359	0.03021
0.55	470,455.4	34.53	4.464	0.030169
0.6	470,506.2	34.57	4.506	0.030119
0.65	473,413.0	34.84	4.566	0.029878
0.7	467,813.5	34.47	4.544	0.029444

**Table 7:** Coefficients obtained from polynomial fitting of parameters in the Arrhenius constitutive model.

Coefficient	*Q*/(J·mol^−1^)	ln*A*	*n*	*α*
X_0_	772,061.7	59.69	8.04	0.04006
X_1_	−6,061,787.8	−488.85	−53.52	−0.30553
X_2_	30,200,000.0	2370.64	225.12	2.10615
X_3_	−63,500,000.0	−4878.19	−401.59	−6.79173
X_4_	59,000,000.0	4438.68	288.92	11.84887
X_5_	−15,400,000.0	−1136.45	0.44	−10.83750
X_6_	−5,440,000.0	−392.12	−68.34	4.05758

**Table 8:** Error evaluation across various models.

Model	AARE/%	RMSE/MPa
Modified J-C model	7.00	5.57
Further-modified J-C model	6.27	4.96
Modified Z-A model	7.24	6.06
Further-modified Z-A model	5.54	4.79
Arrhenius model	9.30	6.40

## Data Availability

The original contributions presented in this study are included in the article. Further inquiries can be directed to the corresponding author.
